# Paying Patients in General Hospitals: An Example from Kimberley

**Published:** 1913-07-12

**Authors:** G. S. Thompson

**Affiliations:** Kimberley, S. Africa


### THE EDITOR'S LETTER-BOX.

### Paying Patients in General Hospitals: An Example from Kimberley.

*To the Editor of* The Hospital.

Sir,—In one of your recent issues I notice a reference to the excellent but long overdue innovation at the Charing Cross Hospital of the system of paying beds. Perhaps it may not be known in England of the excellent system in vogue in several of the hospitals in South Africa, where the sound and proper procedure prevails of dividing hospitals into two parts, paying and non-paying—or, to be more correct, three parts, paying, part-paying, and non-paying. This plan prevails at the Kimberley Hospital. In regard to paying patients there is a graduated scale to meet the varying monetary grades of the population, and we have 5s., 10s., and 15s. beds, according to the accommodation provided. The 5s. per diem patients are placed in a ward; the 10s. ones in a two-bedded room; the 15s. ones in a single room. The paying part of the hospital is open to any practitioner, and in the case of operations times are booked by entering the operation on a slate in the theatre. This system works excellently, and obviously is a great advantage over that which is so commonly in vogue at present, for we have the advantages of the equipment of a large institution as opposed to the reverse in the case of a nursing home, to say nothing of the expense. The part-paying, or intermediate class of patients, are those who, though able to pay a small hospital rate, are unable to meet an additional medical charge. At the Johannesburg Hospital these patients are attended by the honorary member of the visiting staff, this attendance being included in a 3s. 6d. or 5s. daily fee to the hospital.

In regard to the paying patient at this hospital, no charge is made against the medical attendant for anything; for example, there is no theatre fee.

This plan of dealing with paying patients is a most excellent one, which works well in practice. It is obvious that a large institution can equip itself as no nursing home can possibly hope to do or does, whereas the graduated charges will meet every grade of the population in contrast to the often prohibitive charges of nursing homes. Thus this rational system is one that meets the needs of all classes of the community according to their monetary estate. And it seems to me that all hospitals should be divided in this way, so that one may be in a position to treat all patients in a rational, efficient, and satisfactory manner rather than muddling along as is so often the case in England at the present day, to say nothing of the enforced invalidism to which many of the middle classes are liable in surgical conditions owing to the crippling effect of fees difficult or impossible to meet.

Another point which is of importance is that the better class paying patients help to contribute to the maintenance of the free patients; and from this source of revenue at the Johannesburg Hospital I believe about £15,000 a year is obtained. If there are any further points that would interest you and that you would care for information on, I shall be glad to furnish what I can.—Yours truly,

G. S. Thompson, F.R.C.S. (Eng.).

Kimberley, S. Africa,

June 16, 1913.

